# Current Trends, Controversies, and Future Directions in the Evaluation and Management of Superior Canal Dehiscence Syndrome

**DOI:** 10.3389/fneur.2021.638574

**Published:** 2021-04-06

**Authors:** Kristine Elisabeth Eberhard, Divya A. Chari, Hideko Heidi Nakajima, Mads Klokker, Per Cayé-Thomasen, Daniel J. Lee

**Affiliations:** ^1^Department of Otolaryngology, Massachusetts Eye and Ear, Harvard Medical School, Boston, MA, United States; ^2^Copenhagen Hearing and Balance Centre, Department of Otorhinolaryngology, Head and Neck Surgery & Audiology, Copenhagen University Hospital – Rigshospitalet, Copenhagen, Denmark; ^3^Faculty of Health and Medical Sciences, University of Copenhagen, Copenhagen, Denmark

**Keywords:** superior canal dehiscence, semicircular canal dehiscence, third window syndrome, SCD, SSCD, craniotomy, transmastoid, diagnostic

## Abstract

Patients with superior canal dehiscence syndrome (SCDS) can present with a range of auditory and/or vestibular signs and symptoms that are associated with a bony defect of the superior semicircular canal (SSC). Over the past two decades, advances in diagnostic techniques have raised the awareness of SCDS and treatment approaches have been refined to improve patient outcomes. However, a number of challenges remain. First, there is currently no standardized clinical testing algorithm for quantifying the effects of superior canal dehiscence (SCD). SCDS mimics a number of common otologic disorders and established metrics such as supranormal bone conduction thresholds and vestibular evoked myogenic potential (VEMP) measurements; although useful in certain cases, have diagnostic limitations. Second, while high-resolution computed tomography (CT) is the gold standard for the detection of SCD, a bony defect does not always result in signs and symptoms. Third, even when SCD repair is indicated, there is a lack of consensus about nomenclature to describe the SCD, ideal surgical approach, specific repair techniques, and type of materials used. Finally, there is no established algorithm in evaluation of SCDS patients who fail primary repair and may be candidates for revision surgery. Herein, we will discuss both contemporary and emerging diagnostic approaches for patients with SCDS and highlight challenges and controversies in the management of this unique patient cohort.

## Introduction

Superior semicircular canal dehiscence syndrome (SCDS) was first reported by Minor et al. in 1998 (1). The authors described a series of patients with disequilibrium and sound- and pressure-induced vertigo associated with nystagmus in the plane of the superior semicircular canal (SSC). Computed tomography (CT) imaging revealed a bony defect of the SSC. Symptom improvement was observed in patients who underwent surgical plugging of the defect via middle fossa craniotomy. In subsequent years, auditory symptoms, including autophony, amplification of bodily sounds, pulsatile tinnitus, conductive hearing loss, hyperacusis, and aural fullness as well as vestibular symptoms of chronic disequilibrium and sound- and pressure-induced vertigo and oscillopsia became hallmarks of SCDS (2–4).

While in most patients symptoms of SCDS can be tolerated and conservative management is reasonable, some individuals suffering from SCDS report decreased quality of life due to challenges in communicating with those around them and completing activities of daily living (5–7). The health utility value (HUV), a measure of general health-related quality of life, ranges from poor health (0.3), to perfect health (1.0). Indeed, HUV is significantly lower in SCDS patients (0.68) compared to the general U.S. population (0.80) (5). For patients with debilitating symptoms, definitive treatment involves surgical repair of the dehiscence. However, the diagnostic evaluation of patients with suspected SCDS can sometimes be difficult to interpret. Established clinical testing that reveals supranormal bone conduction thresholds, low frequency air-bone gap (ABG) with present acoustic reflexes, low threshold cervical vestibular evoked myogenic potentials (cVEMP) and increased ocular VEMP (oVEMP) amplitudes are useful in guiding management options in symptomatic patients with radiologic superior canal dehiscence (SCD). However, some symptomatic patients do not have findings suggesting a classic third window. Furthermore, while clinicians agree that primary (and revision) surgery is a reasonable option for patients with persistent localizing signs and symptoms, the optimal approach, repair technique and materials are the subject of debate and confusion amongst both providers and patients.

Herein, we will review the pathophysiology and etiology of SCD, present current trends in its diagnosis and management, discuss novel approaches, and finally highlight some of the remaining challenges and controversies. Illustrative cases are provided to complement the literature.

### Pathophysiology

**Figure 1:**
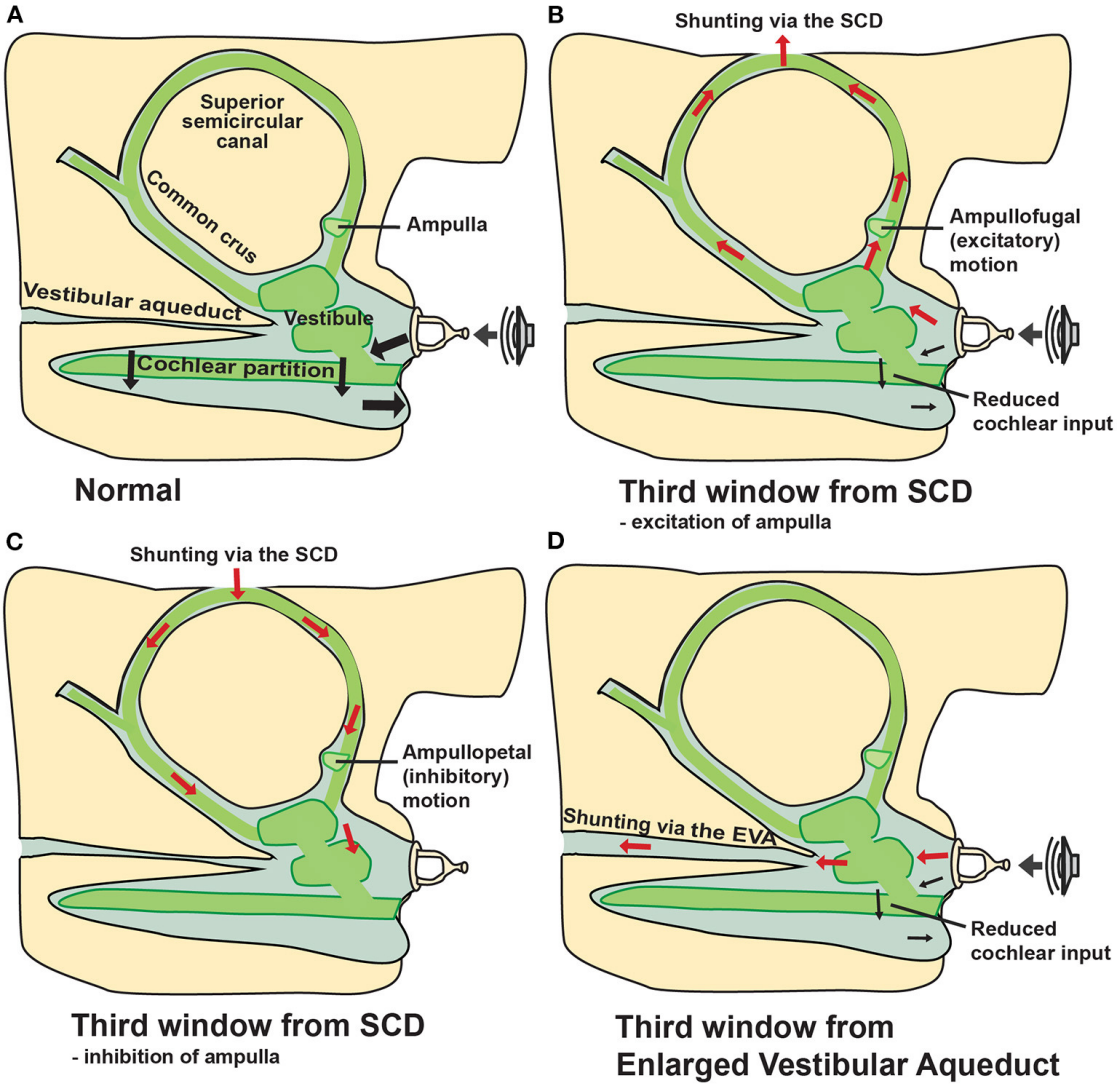
“Third window” mechanism due to SCD and enlarged vestibular aqueduct. Schematic representations illustrate inner ear volume velocity with arrows. **(A)** Normal anatomy allows volume velocity across the cochlear partition from the oval to the round window (two windows). **(B)** Air conducted sound stimulation results in volume velocity from the stapes to be shunted toward the SCD (third window) and away from the cochlea, resulting in increased air-conduction thresholds at low frequencies and/or sound-induced vertigo (Tullio's phenomenon). Positive static pressure in the middle-ear cavity may result in ampullofugal fluid motion exciting the ampulla, resulting in nystagmus (Hennebert sign) and oscillopsia/vertigo (1, 2, 8–12). **(C)** Elevated intracranial pressure from Valsalva against closed glottis (e.g., straining, heaving lifting) may result in ampullopetal endolymphatic fluid motion, inhibition of the ampulla, also leading to nystagmus (Hennebert sign) and oscillopsia/vertigo (1, 8, 11). **(D)** Enlarged vestibular aqueduct (EVA) can also act as a third window, shunting volume velocity away from the cochlear partition and toward the widened vestibular aqueduct (2, 13–15). ^*^Modified from Cheng et al. (16) and Rosowski et al. (8).

Symptoms produced in SCDS are thought to occur by a “third window” phenomenon of the inner ear. In a normal ear, sound is transmitted through the ossicular chain resulting in volume velocity into the cochlea through the oval window and eventually toward the round window (bold arrows in Figure 1A). SCD results in a third mobile window that enables acoustic stimuli at the oval window to dissipate through the vestibular labyrinth, leading to vertigo, and dizziness (Figure 1B). Intracranial pressure changes may also inadvertently stimulate vestibular end organs (Figure 1C) (2, 3, 8, 9). Response to air conduction is reduced resulting in low-frequency hearing loss, and response to bone conduction is increased resulting in hyperacusis, autophony, and amplification of bodily sounds (e.g., hearing eye movements or footfalls). Dural pulsations across the dehiscence are the likely cause of pulsatile tinnitus (a common auditory symptom in SCDS patients). The pathophysiology of SCDS remains incompletely understood, especially with regard to the variability in symptomatology among patients, but remains the focus of a number of research studies (8–11, 17, 18).

Third window lesions may occur in different anatomic locations including the posterior or horizontal semicircular canals, bony vestibule, or the cochlea. An enlarged vestibular aqueduct (EVA) can cause a third window phenomenon in children and adults. A pathologically widened vestibular aqueduct produces a communication between the bony vestibule and intracranial cavity that can result in an ABG and mechanical characteristics similar to that observed in patients with SCDS (Figure 1D) (13). Patients with EVA present with normal hearing thresholds, conductive hearing loss, mixed hearing loss, or sensorineural hearing loss (SNHL) (13–15). Additionally, a dehiscence between the cochlea and the carotid canal, the cochlea and the facial nerve, and between the posterior semicircular canal or the vestibular aqueduct and the jugular bulb/jugular bulb diverticulum have been hypothesized to act as pathological third windows, dissipating acoustic energy away from the cochlear partition (19–22). Third window-like symptoms have also been described in cases of post-traumatic membranous or hypermobile stapes footplate (23).

### Etiology

**Figure 2:**
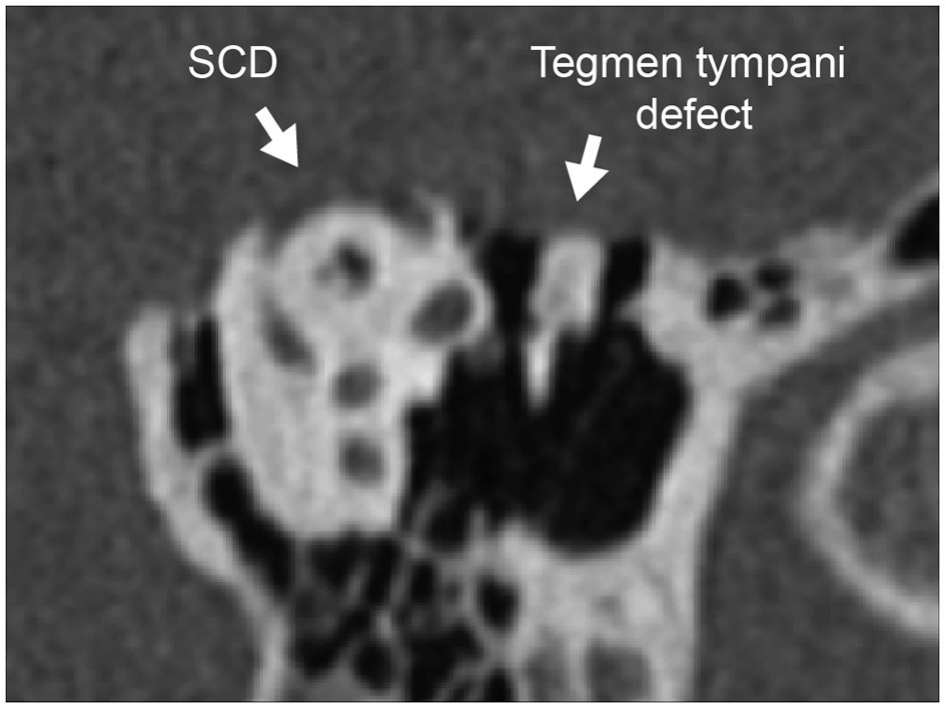
Concomitant tegmen tympani defect with dural herniation. CT of the left ear reformatted to the Pöschl plane demonstrates tegmen tympani bony defect of the skull base and SCD of the arcuate eminence. Herniation of the temporal lobe into the epitympanum can reduce ossicular motion, contributing additionally to the air-bone gap due to SCD and elevating cVEMP threshold (thus masking the lowered cVEMP threshold effect of SCD).

The etiology of SCD is unknown, but two theories have been proposed in the literature: congenital and acquired. The congenital theory of SCD proposes that failure of fetal and postnatal bone development of the temporal bone predisposes to and causes SCD. Proponents of the congenital theory cite temporal bone histopathology studies that show thinning or dehiscence over the superior canal without evidence of bony remodeling (24). Additionally, there is a high prevalence of radiologic SCD in infants, although these findings usually resolve in the first decade of life with the final postnatal bone development (24–27). Some patients with SCD have generalized thin bone throughout the lateral skull base, multiple tegmen defects, and develop SCDs bilaterally, which may further support the congenital theory (24, 28, 29). It has been hypothesized that congenital thin bone of the lateral skull base predisposes a patient to develop SCD due to a second event later in life. For example, head trauma could disrupt the seal over the endosteum or membraneous labyrinth created by the dura, thus resulting in symptomatic SCDS (4, 24, 28, 29). Concomitant tegmen defects are important to recognize as they may alter the findings of audiometric and vestibular testing (Figure 2).

There have been reports of a high prevalence of SCD in patients with a variant of Usher syndrome and overrepresentation of SCD in some families, suggesting that there may be genetic correlates that have not been completely identified (4, 30–32).

The acquired theory of SCD proposes that increased intracranial hypertension and repeated pulsations could degrade the bone overlying the SSC over time. Of note, however, a clear association between intracranial hypertension and SCDS has not been established (33–37). Furthermore, there is not a tendency of obesity among patients who undergo SCD repair (33). Causes of acquired SCD also include: neoplasms such as meningioma (38), vascular malformations (39), chronic osteomyelitis (40), fibrous dysplasia (41), and head trauma with temporal bone fractures (42).

## Diagnostic Advancements and Dilemmas in SCDS

Diagnosing SCDS can be challenging as symptoms vary greatly and may mimic other otologic and neurotologic conditions. The most common symptoms of SCDS are autophony (>50% of patients), amplification of bodily sounds (e.g., hearing eye movements or footfalls, >50% of patients), sound- or pressure-induced vertigo (>50%), aural fullness (>60%), conductive hearing loss (~25–60%), and pulsatile tinnitus (~20–50%) (4, 43–45). Patients also report symptoms of chronic disequilibrium and “brain fog,” that may be related to impaired cognition and a diminished ability to integrate multisensory information (46, 47). The mechanism by which SCD produces such a wide range in symptoms among individuals remains poorly understood (9, 43, 48, 49). A detailed history may reveal symptoms concerning for SCDS and objective findings of the biomechanical effect of SCD, i.e., audiometric testing, VEMP testing, and other novel approaches, can help narrow the differential diagnosis. CT findings of a bony dehiscence over the SSC are diagnostic; however, it is important to recognize that not all individuals with radiologic evidence of dehiscence have relevant symptoms and suffer from SCDS (24, 50).

The diagnostic work-up of SCDS at most centers includes pure tone thresholds to air and masked bone conduction, supranormal bone conduction threshold testing, tympanometry, acoustic reflex testing, cervical, and/or ocular VEMP testing, and CT imaging. In this section, we will (1) discuss the advantages and disadvantages of each of these established testing modalities to narrow the differential diagnosis, and (2) review emerging modalities including wideband acoustic immittance and electrocochleography for evaluating patients with third-window symptoms.

### Audiometric Testing

In the office, bone conduction testing with a 512 Hz tuning fork often lateralizes to the affected (or worse) ear, further supporting the theory that SCD generates a pseudo-conductive hearing loss. Some patients even have the ability to hear (rather than feel) the tuning fork when placed on the malleolus of the ankle (“ankle Weber”) (51). In patients with SCD, pure tone audiometry will often reveal a low frequency (≤1 kHz) ABG, usually in the 15–30 dB range but ABG up to 50 dB has been reported (43, 48, 50, 52, 53). ABG has shown to increase with decreasing frequency, and larger ABG is associated with larger SCD size (9, 12, 48, 54). Furthermore, some SCD patients will have supranormal low frequency (<1 kHz) bone conduction thresholds at −5 to −10 dB HL (8, 11, 18, 50, 53). Low-frequency ABG due to SCD are caused by the combined effects of two separate mechanisms verified by consistency of clinical, temporal bone, and computational modeling data. The low-frequency decrease in air conduction hearing (higher air conduction thresholds) is due to volume velocity shunting via the SCD (Figure 1B) (9, 10). The low-frequency increase in bone conduction hearing (lower bone conduction thresholds) is due to altered inner-ear volume velocities and pressures in response to vibration of the skull and altered mass of the inner ear fluid as determined recently in Guan et al. (11, 18).

The presence of low-frequency conductive hearing loss and other SCDS-related symptoms such as autophony and aural fullness are also seen in patients with otosclerosis, Eustachian tube dysfunction, patulous Eustachian tube, and other middle ear pathologies (43). Acoustic reflex testing and tympanometry are essential to rule out middle-ear pathology or Eustachian tube dysfunction (50, 55). Of note, SCD effects on audiometric, immittance, and VEMP testing may be masked by concomitant middle ear abnormalities or tegmen tympani defects with dural herniation into the middle ear because these conditions affect sound transmission (Figure 2). For example, the dura encroaching into the middle-ear cavity can reduce ossicular motion, thereby increasing the ABG, elevating VEMP thresholds, and decreasing VEMP amplitude. This would obscure SCD-related findings (Figure 2). During impedance measurements such as 226 Hz tympanometry, pulse-synchronous waves have been observed in some SCD patients (56–58).

### Vestibular Evoked Myogenic Potential (VEMP) Testing

VEMP testing assesses the function of the otolith organs of the vestibular periphery by measuring surface electromyography responses to acoustic stimulation. In cVEMP testing, the saccule is stimulated leading to an inhibitory response in the ipsilateral sternocleidomastoid muscle modulated by the inferior vestibular nerve. In oVEMP testing, the utricle is stimulated leading to activation of the contralateral eye muscles. The use of VEMP testing has increased to assess patients with a suspected third window, and many, but not all patients with SCDS, have lowered VEMP thresholds and increased VEMP amplitudes in response to an air-conduction stimulus (depending on selected cutoff values and study populations, cVEMP and oVEMP have a sensitivity and specificity above 70%) (59, 60). A number of studies have demonstrated that the diagnostic utility of cVEMP thresholds and oVEMP amplitudes is better than the diagnostic utility of cVEMP amplitudes and oVEMP thresholds, when using a 500 Hz tone burst or a click stimuli (59, 61–63). However, one challenge is the considerable overlap in VEMP threshold and amplitude between patients with SCDS and healthy, normal ears or asymptomatic ears with radiologic SCD (64).

**Figure 3:**
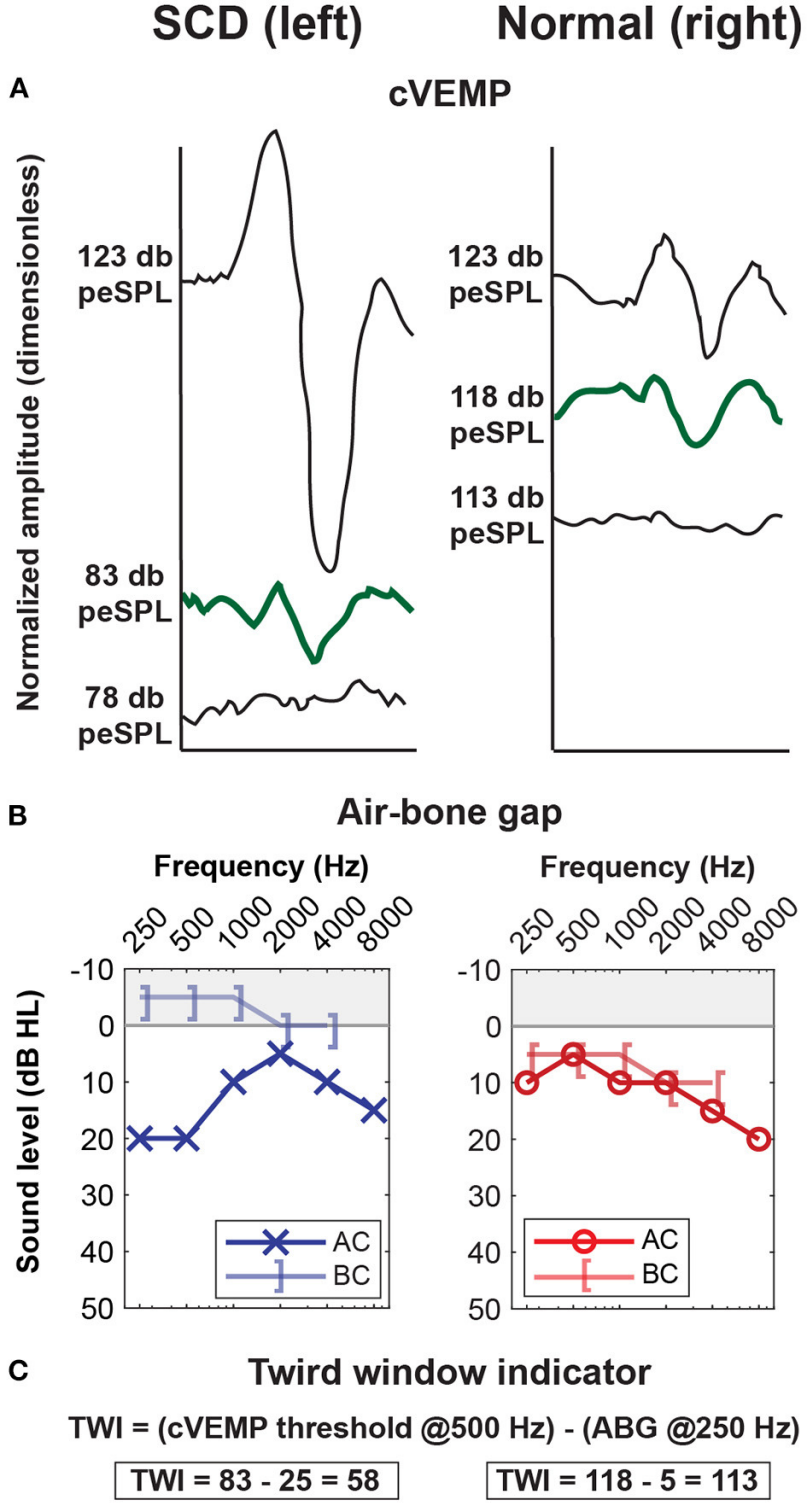
Third Window Indicator (TWI) improves ability to differentiate SCD ears from non-third window ears. Example of an adult patient with symptoms of left-sided SCDS. **(A)** Low threshold cVEMP of 83 dB peSPL [peak sound pressure level, 123 dB peSPL is equivalent to 90 dB HL (65)] at 500 Hz in the left SCDS ear and 118 peSPL in the unaffected right ear. **(B)** Air-bone gap of 25 dB HL at 250 Hz in the left SCDS ear, compared to 5 dB HL in the unaffected right ear. **(C)** The TWI is the difference between the cVEMP threshold at 500 Hz and the air-bone gap at 250 Hz (65). In this case, the TWI is **58** dB for the left SCDS ear, and **113** dB for the unaffected right ear. ^*^Modified from Noij et al. (65).

In an effort to improve the diagnostic accuracy of cVEMP testing, Noij et al. (65) proposed a new diagnostic “third window indicator” (TWI) that combines magnitude of the ABG and cVEMP threshold (Figure 3). The TWI is defined as the absolute difference of the ABG threshold at 250 Hz and the cVEMP threshold at 500 Hz. The authors found that the TWI detected patients with SCDS with greater accuracy compared to ABG and cVEMP thresholds alone (65). Another initiative to improve the diagnostic accuracy of cVEMP was the use 2,000 Hz stimulus instead of the commonly used 500 Hz tone burst. By using a 2,000 Hz tone burst, the authors of the TWI were able to increase the sensitivity and specificity (sensitivity of 92% and specificity of 100% using TWI at 2 kHz vs. 88 and 100% using TWI at 500 Hz), and they furthermore showed that cVEMP amplitude (as a normalized peak-to-peak amplitude) generated superior results (60, 66). High frequency stimuli in oVEMP testing has also shown to be effective in differentiating patients with SCDS from normal controls (one study found sensitivity of 83% and specificity of 93% using 4,000 Hz oVEMP responses vs. 62 and 73% using 500 Hz responses) (67, 68).

Bone-conducted VEMP testing is an alternative approach in cases of concurrent middle ear pathology. oVEMP achieves a higher sensitivity and specificity for both amplitude and threshold testing than cVEMP when a bone-conducted stimulus is used (sensitivity and specificity above 80%) (69).

**Figure 4:**
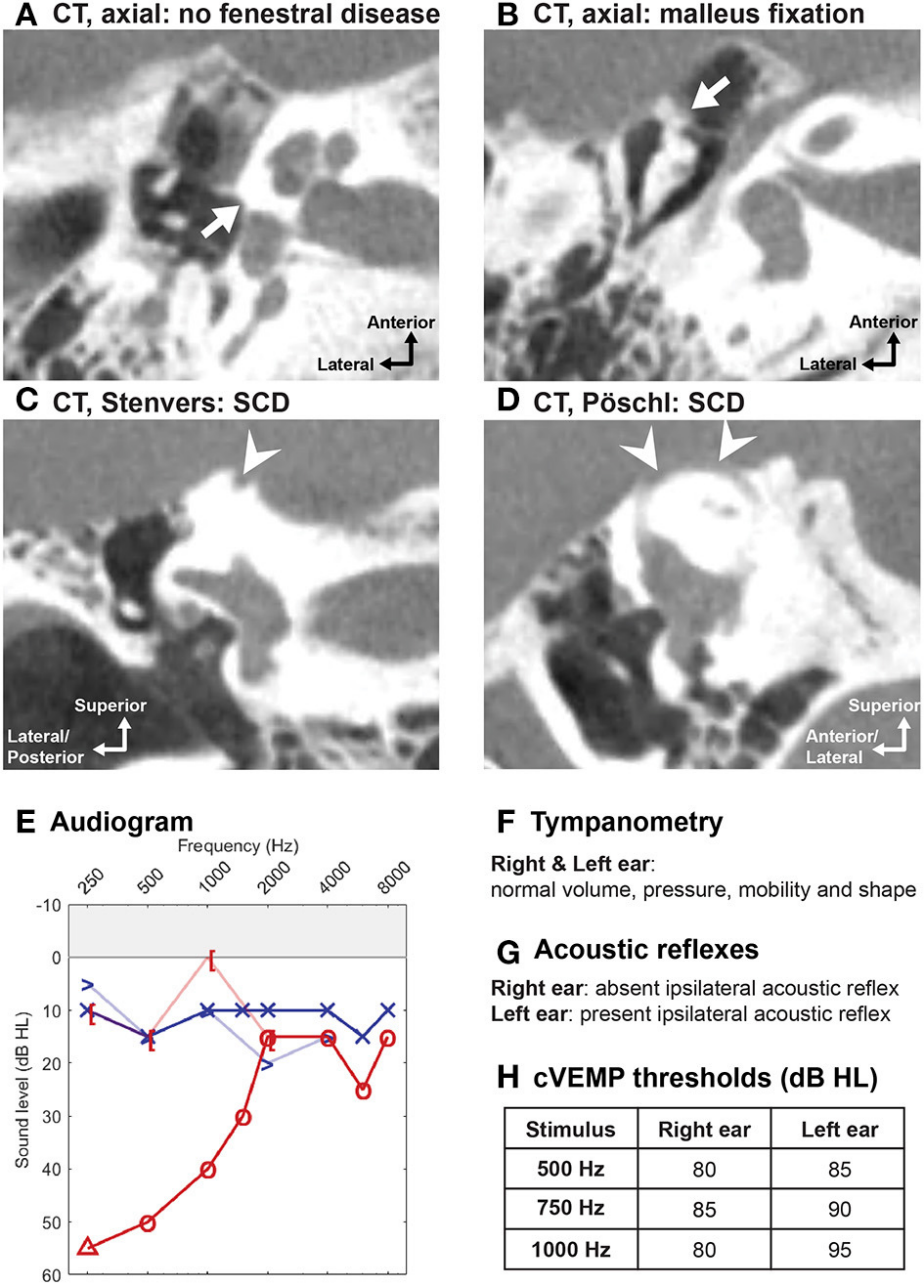
Concomitant SCD and malleus fixation. A 57-year-old woman presented with the sole complaint of hearing loss in the right ear. High-resolution CT scans in right ear: **(A)** absence of fenestral disease [though otosclerosis cannot be excluded or diagnosed on radiologic findings alone (73, 74)] and **(B)** malleus fixation to the anterior epitympanic wall; both **(C)** in the plane of Stenvers and **(D)** in the plane of Pöschl showed SCD at the arcuate eminence. **(E)** Large low-frequency air-bone gap on the right. **(F)** Normal tympanometry bilaterally. **(G)** Absent acoustic reflexes in right ear. **(H)** cVEMP thesholds within normal range (80–85 dB HL) in the right pathological ear, which may reflect a combination of lowered cVEMP thresholds from the SCD and elevated cVEMP thresholds from malleus fixation (64). However, when using the third window indicator and 2,000 Hz stimulus, cVEMP responses were consistent with SCD [Of note, in cases of extensive ossicular chain fixation, cVEMP will be absent (64, 75)]. Given concerns of possible “*unmasking”* of SCD symptoms following ossiculoplasty, conservative management with a hearing aid was recommended for this patient (76).

Despite these diagnostic advancements, there are limitations to the clinical utility of VEMP testing in the evaluation of a patient with suspected SCD. First, VEMP responses assume normal sound transmission through the middle ear, inner ear, otolith organs, and vestibular nerves. Patients with vestibular hypofunction may not demonstrate lowered thresholds or increased amplitudes on VEMP testing of the affected side (70–72), and thus the test may not be used with a high degree of accuracy in this patient population. As vestibular deficits have been observed in some patients following surgical repair of SCD, VEMP testing after surgery can be difficult to interpret. For example, evaluation of patients for revision surgery can be difficult because the thresholds can be elevated for various reasons. VEMP responses are dependent on normal sound transmission to the oval window, which may not be the case if there is middle ear pathology, obscuring the SCD-related changes (Figures 2, 4). Second, VEMP responses decrease with age, although the SCD effect seems to dominate the age effect (60), and conversely, stronger sternocleidomastoid muscle contraction is correlated with larger cVEMP amplitude (60). Third, there is no known association between cVEMP thresholds and severity of auditory or vestibular symptoms (40). Finally, due to lack of standardization in measurement conditions, comparisons of VEMP data across institutions remains challenging and no standard cutoff values for threshold and amplitude exist (60, 70, 77).

### Vestibular Testing

Vestibular function testing, including calorics and vestibular ocular reflexes (VOR) (e.g., video or magnetic scleral search coil head impulse or rotary chair testing), may help exclude other vestibular diagnoses with SCD-mimicking symptoms or global vestibular hypofunction, and provides baseline data for the contralateral ear. For example, patients with contralateral vestibular hypofunction (based on calorics and VEMPs) are at risk for prolonged recovery following surgical repair (78). Vestibular testing is critical in the evaluation of patients for revision SCD surgery. For example, caloric testing will assay the residual function of the *superior* vestibular nerve (cVEMPs measure *inferior* vestibular nerve function) in the operated ear and provides baseline data on the function of the contralateral ear. In patients with bilateral SCD, evaluation of residual vestibular function of the operated ear is useful prior to consideration for surgery in the second ear (79).

A vertical torsional nystagmus (in the plane of the SSC) elicited by sound and/or pressure stimuli (e.g., using pneumatic otoscopy or tragal pressure) can be examined using Frenzel lenses, magnetic scleral search coil, or video nystagmography (1, 78, 80). Indeed not all patients have sound- and pressure-induced vertigo or nystagmus, and even in patients with subjective vertigo to sound and pressure stimuli, a nystagmus may not be detected (of note, there is limited literature on the prevalence of this finding in SCDS patients) (1, 78, 80).

In some patients with a large dehiscence, the VOR response to e.g., head impulse testing may be reduced compared to normal (one study suggested relevance for SCDs ≥5 mm) (80–82). This inverse relationship between SCD size and VOR gain could be explained by “auto-plugging”: in ears with a large dehiscence dura may herniate through the dehiscence, compress the membranous labyrinth, and thus impede endolymph flow during head rotation (80–82). In patients who experience sound- and/or pressure-induced vertigo this “auto-plugging” may be incomplete or intermittent. This relationship has implications when interpreting VOR in the presence of a large SCD.

### Wideband Acoustic Immittance (WAI)

**Figure 5:**
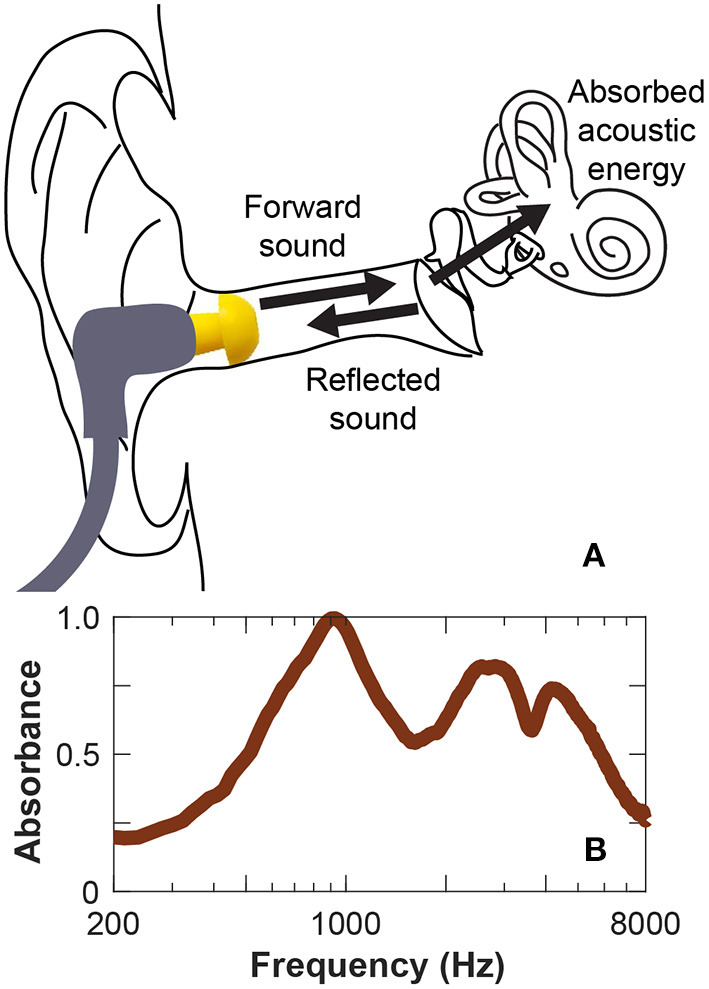
Wideband acoustic immittance testing. Wideband acoustic immittance is a non-invasive measurement that can quantify the acoustic influence of a third window on the impedance of the ear. **(A)** *Absorbance*, or absorbed acoustic energy is measured by presenting acoustic stimulus to the ear canal and measuring reflected sound. **(B)** SCD increases absorbance where a characteristic peak in absorbance around 1 kHz is observed (83, 84).

WAI is a non-invasive measure of the mechano-acoustic impedance of the middle and inner ear. While standard tympanometry uses a single frequency acoustic stimulus, WAI measures function across a range of acoustic frequencies. Wideband tympanometry is WAI measured at different static pressures. One of the most commonly computed metric of WAI is *absorbance*, a measure of the power ratio of reflected sound from the eardrum and the forward sound stimulus presented at the ear canal (Figure 5A) (85).

SCD, a mechanical pathology, decreases inner ear impedance, resulting in a peak in absorbance around 1 kHz (Figure 5B). Thus WAI is a potential screening tool for SCDS (83, 84). Improved diagnostic accuracy has been achieved with WAI by using advanced analytical techniques such as structure-based computational modeling and machine learning algorithms (86). These methods also serve to automate the diagnostic capability of WAI, especially if combined with audiometric and/or other measurements. Limitation of WAI is that it measures the sum of the impedances of the ear, thus is sensitive to biomechanical effects of the middle ear. For example, a hypermobile tympanic membrane or the presence of ossicular fixation will affect the WAI.

Because SCD is a mechanical pathology affecting the acoustics of the inner ear, WAI may serve an important role in the evaluation of patients with residual signs and symptoms following primary SCD repair. Following surgical repair of SCD where the dehiscence is successfully sealed, the SCD-related changes in WAI will disappear (86). Unlike VEMP measurements that require a functional inferior vestibular nerve pathway, WAI is a mechanical measure of inner ear impendance and may be useful in the assessment of a revision SCD candidate who may have vestibular dysfunction following primary repair.

To date, only few institutions use WAI for diagnosing etiologies of conductive hearing loss. One barrier to widespread use of WAI is due to the complexity of data interpretation. As additional tools are developed to analyze the data and automate diagnoses, WAI may become more widely used.

### Electrocochleography (ECochG)

ECochG measures the electric potentials of the cochlea and the cochlear nerve in response to sound stimulation. The electrode is placed either on the surface of the tympanic membrane or in the middle-ear cavity on the promontory of the cochlea or near the round window during a transcanal approach. Various electrical phenomena have been observed: summating potential (SP) reflects direct current (DC), cochlear microphonic reflects the alternating current (AC), while action potential (AP) waveform reflects auditory nerve activity. Historically, ECochG was used to evaluate patients with suspected Menières disease. When SCD is present, the relative static pressure of perilymph in scala vestibuli and scala tympani is reduced compared to static pressures in the endolymph of scala media, thus mimicking the conditions of endolymphatic hydrops (87). These hydrostatic changes of the inner ear are thought to lead to similar ECochG measurements of elevated SP amplitude and SP to AP amplitude ratio (87, 88).

A number of studies have shown that SP/AP amplitude ratio in most cases can be used to differentiate between SCDS ears and normal or unaffected ears (sensitivity and specificity > 70%) (87, 88). The elevated SP/AP amplitude ratio reverses following surgical plugging of the affected canal (three studies, total of 18 patients with elevated SP/AP ratio preoperatively, 17/18 patients with normalized SP/AP postoperatively, 1/18 with SNHL following surgery, four patients potentially contributed to two studies) (87–89).

ECochG has been used intraoperatively to 1) monitor hearing during SCD repair and 2) confirm canal occlusion. An immediate reduction in the SP/AP amplitude ratio is seen when the canal is occluded (statistically significant, total of 42 ears) (87, 90). While it appears that ECochG may provide intraoperative feedback following canal occlusion, intraoperative ECochG monitoring has not yet been correlated with postoperative symptom resolution or hearing preservation (90).

### Imaging—CT Classification of SCD

**Figure 6:**
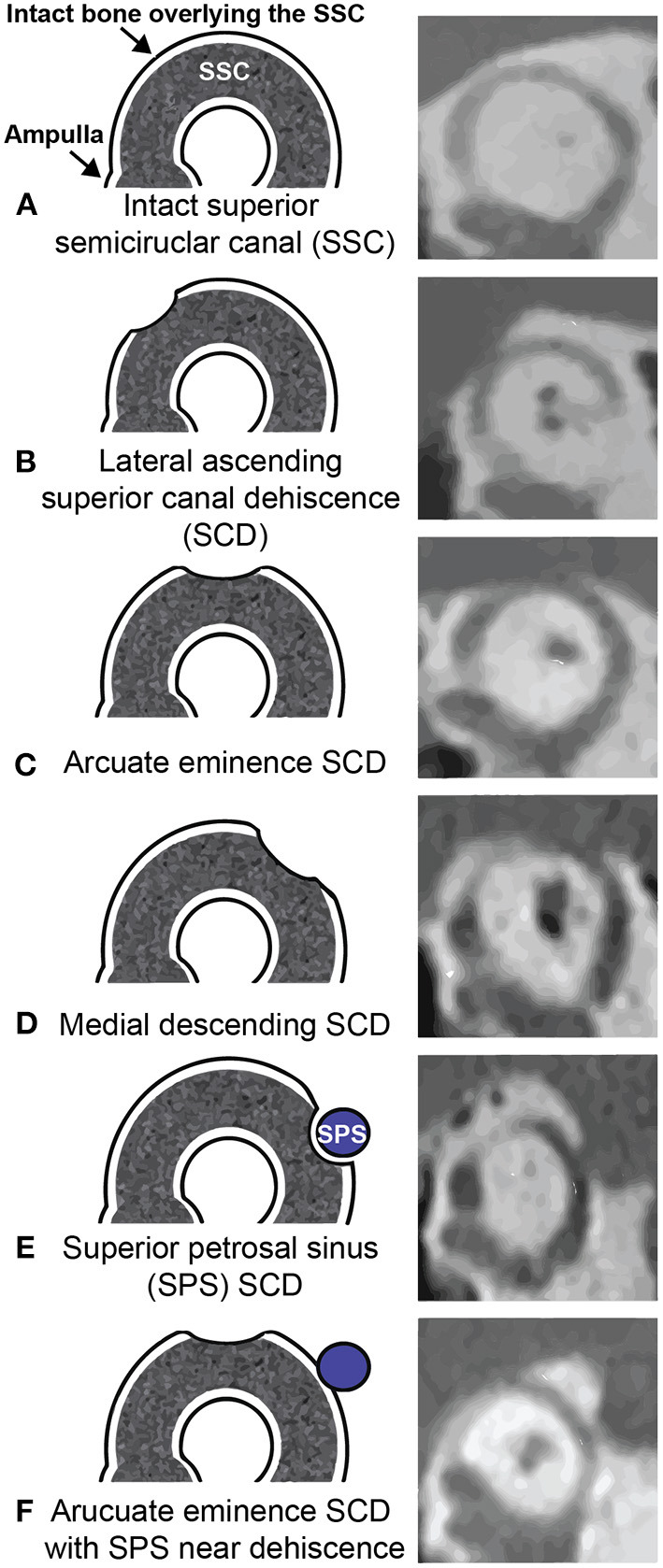
Massachusetts Eye and Ear CT classification of SCD. **Left column (A–F)** illustrates the location of superior semicircular canal defect corresponding to the **right column (A-F)** CT images in the Pöschl plane. SCD size and location are important parameters to consider for surgical planning. ^*^Adapted with permission from Wolters Kluwer Health, Inc.: Lookabaugh et al. (91).

The gold standard for the radiologic diagnosis of SCD is high-resolution CT. Our group has proposed a CT classification scheme to standardize the description of the dehiscence along the SSC and aid in surgical planning (Figure 6) (91). The approach for SCD repair is influenced by the location of the bony defect and its relationship to surrounding tegmen topography. In an analysis of 316 ears with SCDS, the most common location for SCD (on CT) was the arcuate eminence (59%), followed by medial descending limb (29%), lateral ascending limb (8%), and descending limb associated with the superior petrosal sinus (4%). In rare cases, bony defects at two separate locations are observed (<1%) (91).

### Imaging—Improving CT Diagnosis of SCD

Due to the effect of volume averaging, routine temporal bone imaging may falsely detect a dehiscence, particularly when the bone overlying the canal is thin (92–94). Several methods have been developed to detect thin bone and dehiscence accurately: (1) decreasing the collimation thickness from 1 to 0.5 mm; (2) reformatting the images from the coronal and axial planes to the plane of the superior canal (Pöschl) and orthogonal to it (Stenvers); (3) assessing density of pixels along the roof of the superior canal to account for volume averaging; and (4) utilizing gray-scale inversion (invert function) to improve visualization and contrast of subtle changes (95–98). It has also been suggested to set a criterion of bony dehiscence in at least two consecutive CT slices (91).

Improvements in imaging modalities have increased the accuracy of detecting a bony defect of the superior canal. Multislice CT (MSCT) scans are commonly used to evaluate patients with a suspected third window but newer approaches using flat panel detector (cone-beam) CT (FPCT) are more accurate (linear correlation for FPCT estimates of SCD length and surgical measurement, *R*^2^ = 0.93; linear correlation for MSCT estimate and surgical measurement, *R*^2^ = 0.28 with MSCT tending to overestimate SCD length) (99). However, a radiologic dehiscence may be an incidental finding without clinically relevant symptoms. It is hypothesized that in these patients, the dura creates a tight seal above the canal, which protects from the acoustic impedance changes caused by the dehiscence (24). Radiologic canal dehiscence in the absence of symptomatic SCDS does not warrant surgical intervention (4, 78). SCDS must be diagnosed based on localizing signs and symptoms and objective testing, i.e., audiometric and VEMP testing (44, 78). Sole evidence of dehiscence on imaging is insufficient to make a diagnosis of SCDS or surgical intervention.

### Role of MRI in the Initial Evaluation of Patients With SCD

**Figure 7:**
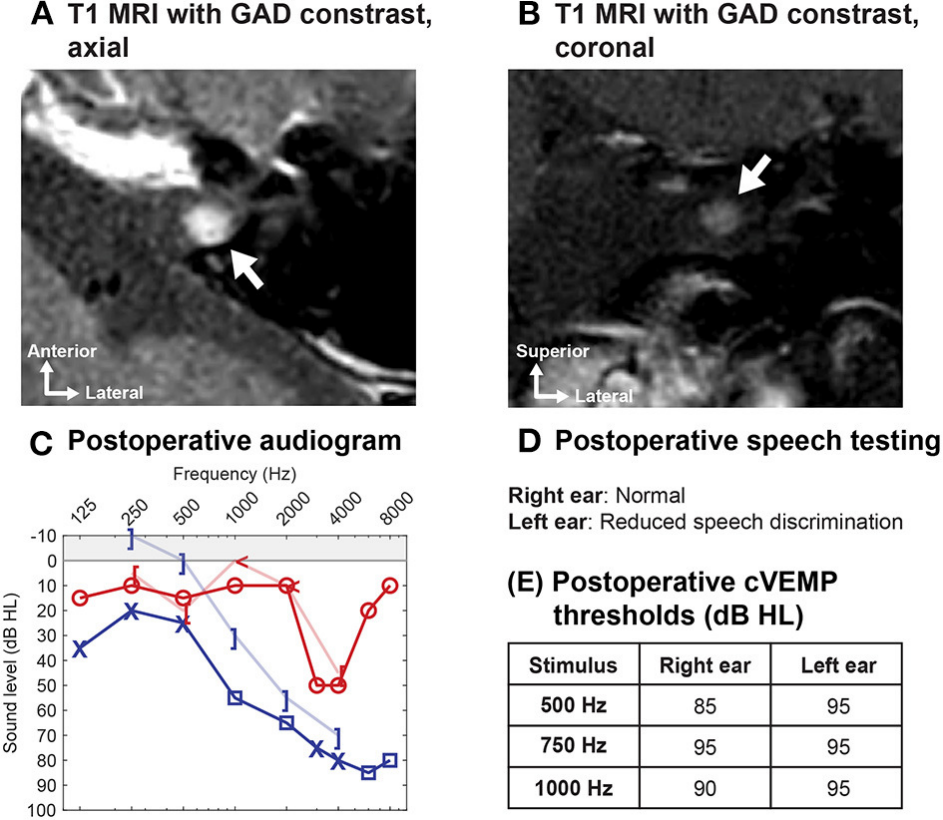
Utility of MRI in preoperative evaluation of SCD. A patient who underwent transmastoid SCD repair for symptomatic left ear SCDS reported progressive hearing loss several months after surgery. **(A,B)** Postoperative high-resolution temporal bone MRI with gadolinium (GAD) contrast revealed a focal enhancing lesion of the left internal auditory canal, consistent with a possible superior vestibular nerve schwannoma (arrow). **(C,D)** Audiogram 1 year after SCD repair shows left-sided mixed hearing loss with poor speech discrimination score (progression compared to preoperatively). **(E)** The cVEMP thresholds show preservation of function in the operated left ear. As this small tumor involved the superior vestibular nerve, cVEMP thresholds (driven by the inferior vestibular nerve) would be preserved (or elevated due to SCD surgery). SCD surgery would *not* have been offered if the diagnosis of schwannoma was made preoperatively in either ear, underscoring the importance of a contrast-enhanced high-resolution temporal bone MRI in the workup of SCD. In rare cases, a lateral skull base tumor (e.g., meningioma) found on MRI has been associated with erosion into the superior semicircular canal and SCD symptoms (38).

Magnetic resonance imaging (MRI) is used increasingly in the preoperative assessment of patients with SCDS and provides complimentary imaging data to CT. Used as a sole modality in the assessment of a suspected third window, high-resolution T2-weighted temporal bone MRI (CISS, FIESTA, etc.) can *exclude* the presence of SCD (and avoid the need for CT in some cases) but it may also falsely detect canal dehiscence in ears with thin bone overlying the canal as seen on CT (e.g., in two studies, 20–39% of ears with SCD seen on MRI had bony covering of the SSC on CT) (100, 101). MRI is important to rule out associated intracranial pathology that may influence surgical decision making. For example, MRI can exclude the presence of a temporal encephalocele, vestibular schwannoma, vascular malformation, or a lateral skull base meningioma (a rare cause of SCD) (Figure 7) (38, 40).

### Utility of Temporal Bone MRI in Patients Who Are Candidates for Revision SCD Repair

**Figure 8:**
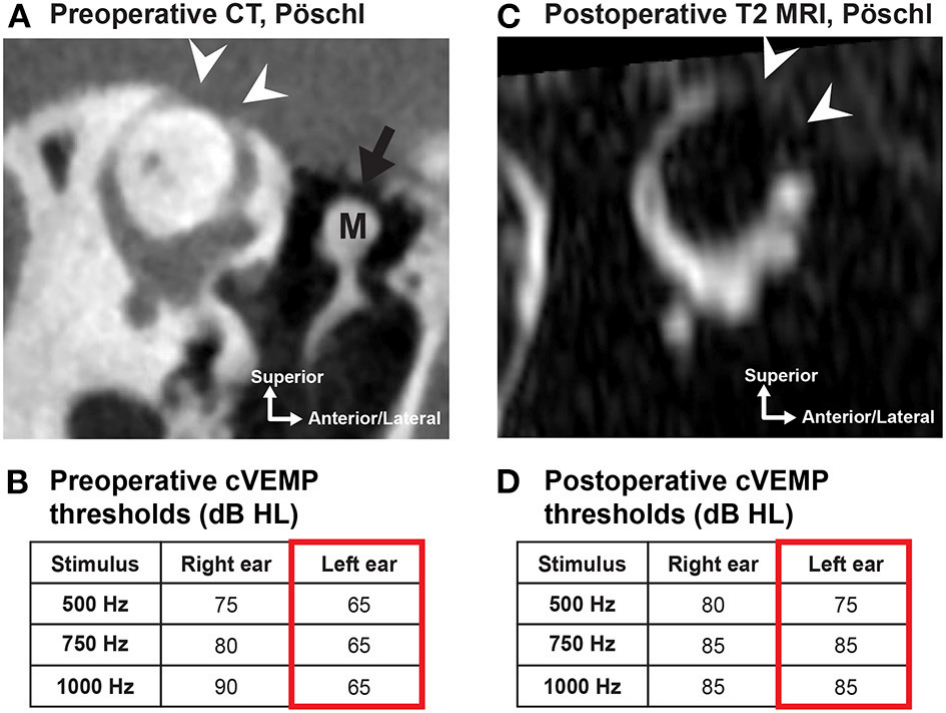
MRI can assess the extent of repair following SCD surgery. **(A)** Preoperative high-resolution CT in the Pöschl plane in a 56-year old male patient with left-sided SCDS. Arcuate eminence defect (white arrowheads). Tegmen tympani dehiscence (black arrow) without dural contact to the malleus (M). **(B)** Preoperative cVEMP thresholds of the left ear at 500, 750, and 1,000 Hz were *lower* (mean: 65 dB HL) than the asymptomatic right ear (mean: 82 dB HL). **(C)** Postoperative T2-weighted MRI in the Pöschl plane illustrates a *fluid void* (no fluid signal) extending beyond the original SCD region seen on preoperative CT, confirming occlusion of the SCD following uneventful surgery with middle cranial fossa approach. **(D)** Postoperative cVEMP thresholds show *elevation* (normalization) in the operated ear (mean: 82 dB HL). Patient also had resolution of primary complaint.

MRI is a valuable diagnostic modality in the evaluation of patients considering revision surgery for SCDS. Postoperative CT provides little information on the extent of the SCD repair because most materials used to repair SCD (e.g., bone wax, fascia, cartilage) are not radio-opaque (except when bone chips or bone cement are used to plug or resurface/cap the canal). However, high-resolution T2-weighted MRI formatted to the plane of Pöschl can be used to evaluate the extent of surgical occlusion and identify any residual defects by assessing the fluid void (lack of fluid flow) within the SSC (a proxy for extent of SCD plugging) (Figure 8) (102–104). By using both MRI and CT, the fluid void on Pöschl MRI views can be compared to the location and length of the bony dehiscence seen on Pöschl CT to determine if revision surgery may be indicated (by using this method, a residual defect was found in ~6/9 patients with symptom recurrence vs. 1/4 patients with complete symptom resolution following SCD occlusion repair) (102).

The posterior-medial (descending) limb of the SSC is the most common region with residual defects following middle fossa craniotomy (5/9 ears with residual defect in posterior-medial limb vs. 3/9 ears with residual defect in anterior-lateral limb) (102). If the Pöschl MRI demonstrates a fluid void that does not fully encompass the bony defect on Pöschl CT (consistent with insufficient occlusion and persistent defect), a transmastoid approach to occlude the remaining limb may be indicated (102, 103). One should be aware that aggressive repair with autologous or non-autologous repair materials at the antero-lateral (ascending) limb toward the ampullated end of the SSC could injure the neuroepithelium of the ampulla (102).

## Surgical Management: Considerations and Controversies

**Figure 9:**
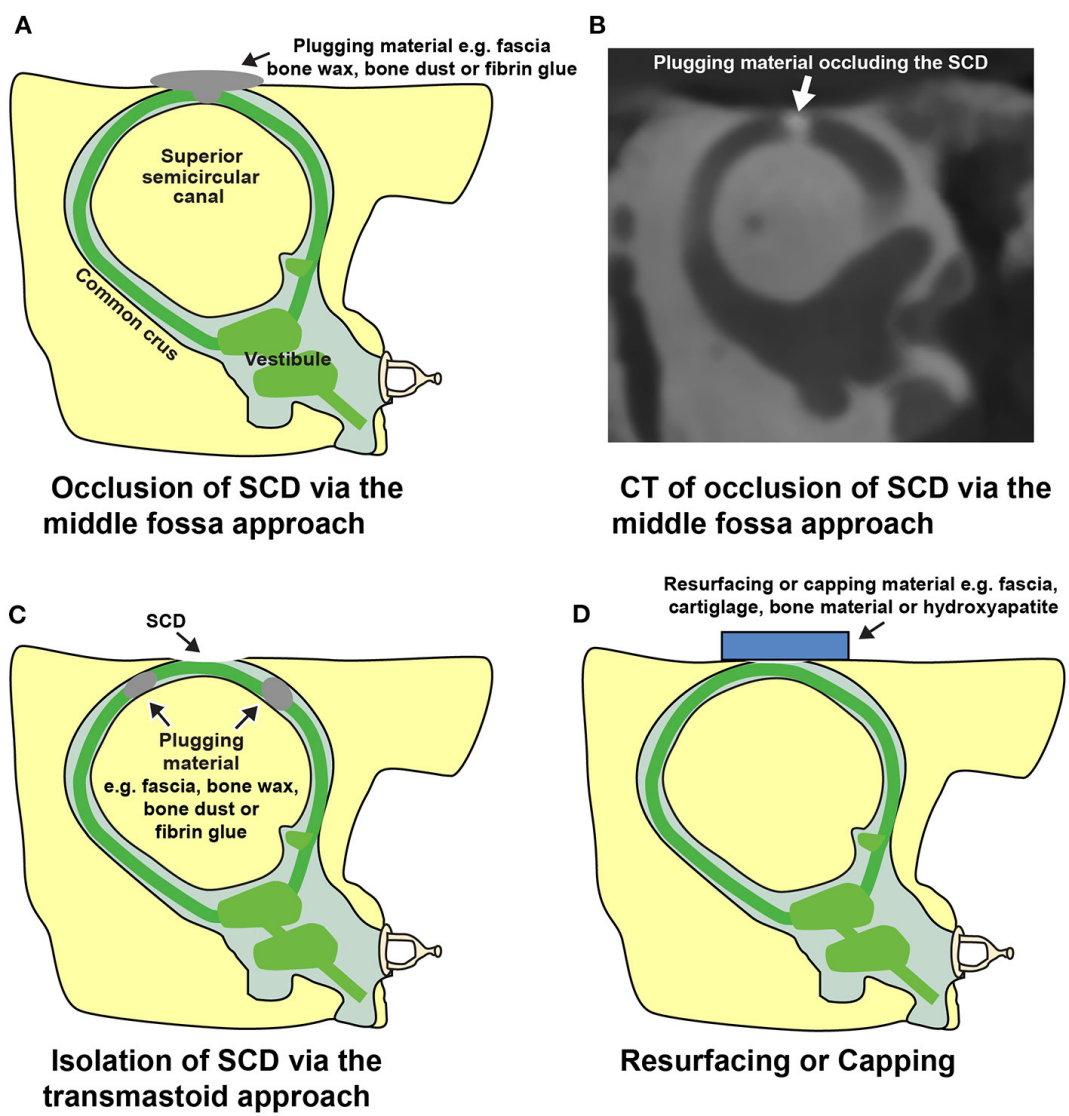
Illustrations depicting surgical repairs of SCD. **(A)** Plugging or occlusion of an arcuate eminence defect via middle fossa craniotomy approach. **(B)** CT image in the Pöschl view following repair. Occlusion was performed in a cadaveric temporal bone model of SCD using contrast-infused surgical bone wax. **(C)** Transmastoid approach for repair of SCD. A labyrinthotomy is created in the ascending and descending limbs of the superior semicircular canal and plugged to isolate the SCD. **(D)** Resurfacing or capping an arcuate eminence defect. This approach attempts to create a seal without occluding the superior semicircular canal lumen. ^*^Modified from Cheng et al. (16).

As there are no known effective medical therapies for SCDS, surgery remains a reasonable treatment option for patients with intractable vestibular and/or auditory symptoms that localize to the side of the radiologic SCD. The goal of surgery is to reduce or eliminate the third mobile window phenomenon. Durable and effective SCD repair must create a watertight seal at the dehiscence site. This is most commonly achieved by occluding the SCD by direct exposure and repair via middle fossa craniotomy (MFC) (Figures 9A,B) or directly or indirectly using a transmastoid approach (Figure 9C). A resurfacing or capping technique can be used as well from either surgical corridor (Figure 9D) but is associated with a higher failure rate (44, 50, 105, 106). However, there remains a relative lack of consensus in the literature about the optimal surgical technique (106, 107).

### Surgical Outcomes

Resolution of the chief complaint (either vestibular or auditory) is observed in most patients who undergo SCD repair (33/33 patients) (45). However, mechanically-induced symptoms such as low-frequency conductive hearing loss, autophony, pulsatile tinnitus, and sound- and pressure-induced vertigo appear to resolve more readily compared to symptoms of headaches, chronic disequilibrium, and brain fog (5, 45, 108–111) (i.e., three studies with a total of 124 patients reported postoperative resolution of symptoms of autophony, pulsatile tinnitus and sound- and pressure-induced vertigo in the range of 73–100%, compared to 63–95% for general disequilibrium and aural fullness) (45, 109, 110).

The reported risk of major complications following SCD surgery is low (107, 110, 112, 113). The most common complications include SNHL [profound SNHL ~2.5%;(112) mild SNHL ~25%(114)] and balance dysfunction [studies report that 39–80% of patients have balance dysfunction in the first postoperative week with resolution in more than half (115, 116), and that transient room-spinning vertigo due to benign paroxysmal positional vertigo (BPPV) is seen in 4.5–24%(107, 110, 112, 113, 117)]. Rare complications include facial nerve paralysis (reported following MFC), epidural hematoma (reported following MFC), dural tear (reported following both approaches) and surgical site infection (reported following both), and overall the rare complication rate is <1.5% (45, 107, 109, 112).

Postoperative audiometric, VEMP, and vestibular testing are routine measures to assess auditory and vestibular function following surgery. Reversal of SCD effects on audiometric and VEMP testing are observed: (1) Closure of the ABG (mean preoperative low-frequency ABG of 16 dB vs. 8 dB postoperatively, 43 ears) (114); (2) normalization of supranormal bone conduction thresholds (median preoperative thresholds of −5 dB HL vs. +5 dB HL postoperatively, 43 ears) (114); and (3) normalization of cVEMP thresholds and oVEMP amplitudes (significant among 12 subjects) (118) have been associated with successful symptom resolution. Several validated questionnaires have been used to quantify pre- and postoperative SCD signs and symptoms, including the Autophony Index (119), Dizziness Handicap Inventory (DHI) (120), Hearing Handicap Inventory (HHI) (121, 122), and Tinnitus Handicap Inventory (123). As mentioned previously, high-resolution T2-weighted MRI (with Pöschl reformats) is useful to evaluate the extent of canal occlusion following surgery and identify any residual defects that can be associated with residual symptoms (102–104). Figure 8 highlights the correlation of radiologic confirmation of SCD repair with reversal of diagnostic indicators in a patient with durable symptom control after surgery.

### Risk of Postoperative Sensorineural Hearing Loss

Transient SNHL postoperatively has been reported (113, 114, 124) and can accompany labyrinthine hypofunction (114, 124). One study (43 patients) reported that about 50% of surgical SCD patients had at least a mild SNHL measured at 7–10 days after surgery. Bone conduction thresholds tend to increase: at low frequencies bone conduction thresholds normalize from supranormal or low thresholds, and at higher frequencies thresholds may increase above normal range (114). About 25 % of patients treated with systemic steroids for 10–14 days continue to have some SNHL (>1 month) (114).

Persistent mild SNHL following primary surgical repair of dehiscence is not uncommon and typically manifests as a high frequency loss (78, 107, 114, 125) i.e., two studies (43+34 patients) reported a mean 10 dB elevation of air conduction thresholds at 4–8 kHz (significant), which did not affect speech discrimination, and mild SNHL in ~25% (114, 125). Postoperative moderate to profound SNHL is rare (44, 112, 114), likely around 2.5% (6/242 patients) (112), and can present in a delayed fashion (e.g., 1 week postoperatively) (44, 126). Some reports have indicated an increased risk of SNHL with multiple inner ear surgeries (i.e., revision SCD surgery or SCD surgery following stapedotomy) (103, 112, 114, 127). The largest study reported profound SNHL in 2.3% (5/220 patients) of primary repair cases and in 4.5% of revision repairs (112), while another study showed (though not significant) larger decrease in speech discrimination and pure tone average thresholds among revision cases (21 patients) than primary repairs (27 patients) (103). In summary, in the majority of patients undergoing primary repair, hearing thresholds remain stable or are minimally affected, and word recognition scores are unchanged (110, 113, 114, 127, 128).

Some centers perform intraoperative ECochG and auditory brainstem responses (ABR). However, neither ECochG SP/AP amplitude nor ABR latency appear to successfully predict postoperative hearing outcome (90). Conversely, intraoperative ECochG monitoring in which an instantaneous SP/AP amplitude reduction is achieved upon repair of the dehiscence may provide an objective measurement of successful repair (87, 90).

Cochlear implantation in the presence of SCD does not appear to unmask or worsen SCD symptoms. However, patients with radiologic dehiscence or SCDS had worse speech perception than patients without canal dehiscence (129).

### Risk of Dizziness and Balance Impairment After SCD Repair

Vestibular impairment in the acute postoperative setting is commonly reported (39–80% of patients) (115, 116). The mechanism of this phenomenon and of transient SNHL is unknown. It is hypothesized to be related to the surgical trauma. Potential mechanisms include: (1) labyrinthitis, (2) loss of perilymph disturbing labyrinthine function, (3) compression of the membranous labyrinth with displacement of endolymph causing a “hydrops-like” condition, and (4) membranous labyrinth tears allowing ion exchange between the otherwise confined compartments (111, 115, 116, 130, 131). Additionally, a reduction of SSC function from occlusion repair may cause acute vestibular impairment. In most cases, vestibular impairment resolves or the patient is able to compensate for loss of function within several months [one study found resolution in 70%(116)] (78, 115, 116). Patients with ipsilateral vestibular hypofunction and concomitant SNHL may suffer from labyrinthitis, and treatment with steroids and vestibular therapy can be beneficial [one study reported 2/19 patients with global vestibular hypofunction (81), and another reported 3/16 ears with postoperative SNHL and vestibular hypofunction that resolved on a steroid taper (124)]. Vestibular examination within the first postoperative week will likely show spontaneous and/or post-head-shaking nystagmus (90% of patients), and often as an irritative nystagmus indicating increased excitability (70% of patients), alternatively as a paralytic nystagmus indicating hypofunction (only data on patients with repair by occlusion technique) (115). VOR testing following surgical repair by occlusion will most often show reduced function of the SSC (4/4 and 4/7 patients with reduced VOR gain) (130, 131) and may also show decreased function of the ipsilateral posterior and horizontal semicircular canals (116, 130). This is consistent with vestibular impairment in the acute postoperative setting. One study suggested that over time (months), VOR gain for the SSC can normalize to preoperative values (11 patients) (131), whereas other studies show sustained reduction and only partial improvement in SSC function (19, 5 and 10 patients) (81, 130, 132). Reduced SSC function alone can likely not explain cases of prolonged vestibular impairment.

Prolonged vestibular impairment is common among patients with a concomitant migraine diagnosis or with bilateral SCDS (one study found prolonged vestibular impairment in 13/13 migraine patients vs. 8/25 non-migraine patients) (133), likely due to the more generalized vestibular impairment prior to surgery and a reduced ability of central compensation (45, 133). Patients with bilateral SCD repair are also at risk of persistent oscillopsia, suggesting increased risk of chronic oscillopsia in patients with contralateral vestibular hypofunction (2/4 patients) (79). The ipsilateral horizontal and posterior semicircular canal impairment observed in some patients in the acute postoperative setting is often normalized at long term follow-up (months) (81, 116, 130–132), though sustained reduction of posterior semicircular canal function is seen (81, 130). This stresses the importance of vestibular testing prior to second-sided surgery. Prolonged balance dysfunction may also be exacerbated by episodic BPPV, which occurs not infrequently following SCD repair (4.5–24%, two studies with 242 and 84 subjects, respectively) (112, 117).

### Middle Fossa Craniotomy (MFC) and Transmastoid Approaches

The original publication on SCDS by Minor et al. described repair by MFC approach (Figures 9A,B) (1). They used a “plugging” technique to achieve resolution of symptoms but “resurfacing” and “capping” techniques have also been described. The repair techniques are discussed in greater detail in subsequent sections.

**Figure 10:**
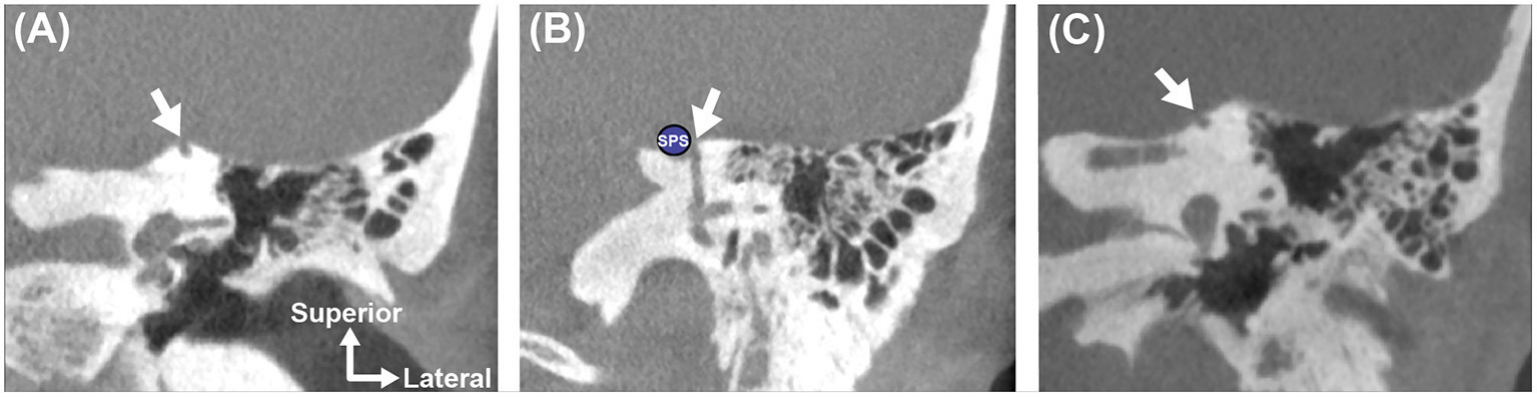
Anatomic location of SCD influences surgical approach and can predict challenging dissection. Coronal high-resolution CT imaging highlights three distinct left ear superior canal defects. **(A)** SCD involving the *arcuate eminence*. This defect is easily accessed and directly visualized via the middle fossa craniotomy approach. **(B)** SCD involving the superior petrosal sinus (SPS). This defect should be repaired using a transmastoid approach with plugging of the ascending and descending limbs of the superior semicircular canal to isolate the defect around the SPS and avoid disrupting the sinus (134). **(C)** SCD involving the *medial* surface of the arcuate eminence along a downsloping tegmen. This defect may be difficult to visualize with a microscopic-assisted middle fossa craniotomy approach unless a large craniotomy and significant brain retraction are performed. To safely identify and repair this type of defect, an endoscopic-assisted middle fossa craniotomy (135) or transmastoid approach may be used.

As an alternative to the MFC approach, the SSC may also be accessed either directly or indirectly by transmastoid approach (Figure 9C) (109, 113). The selection of surgical approach is often influenced by the anatomy surrounding the defect and the experience of the surgeon. Lookabaugh et al. (91), who proposed a CT classification of SCD (Figure 6), suggested that the location of the dehiscence can be used to determine surgical approach (91). For example, an arcuate eminence defect (59% of SCDS) may be safely reached using the MFC approach (Figure 10A), and a contracted mastoid or a low-lying tegmen are suited for an MFC. In contrast, a bony dehiscence along the posterior-medial (descending) limb of the superior canal (29% of SCDs), and associated with the superior petrosal sinus (4% of SCDs) are ideally repaired using a transmastoid corridor to avoid direct manipulation of a skull base venous sinus via MFC (Figure 10B) (these defects often do not have a low lying tegmen or associated skull base bony defects).

An important advantage of the MFC is that it enables the surgeon to directly visualize the dehiscence and associated tegmen defects, but may carry a slightly increased risk of cerebrospinal fluid (CSF) leak, stroke and other complications related to craniotomy (45, 112, 136, 137). The transmastoid approach is less invasive than the MFC approach and may be performed in the outpatient setting (132, 134).

Skull base endoscopy using a 0 degree or angled Hopkins rod telescope can be a valuable adjunct to traditional line of sight microscopic-assisted (or exoscopic) SCD repair methods when attempting to visualize “hidden” superior canal defects (135, 138–140). In patients where the arcuate eminence defect is associated with a downsloping tegmen, the microscopic view is limited, necessitating a large craniotomy or extensive temporal lobe retraction (135, 139). An endoscope (e.g., angled) can be utilized through a smaller MFC or keyhole craniotomy to provide superior transillumination of the skull base and identification and characterization of the bony dehiscence (135, 138–140). An example of a defect that is located toward the posterior limb of the superior canal along a downsloping tegmen is shown in Figure 10C. Endoscopic transillumination of a blue-lined dehiscence case has been described, suggesting that locating the bony defect may be more facile and accurate with the endoscope (135, 139).

Due to high variability among studies, it is currently difficult to determine if a specific surgical approach is associated with better outcomes.

### Canal Plugging and Resurfacing/Capping

To restore labyrinthine biomechanics and reverse the third mobile window effects, a tight fluid seal must be created (12, 141). Several groups have reported plugging of the SSC to obtain a tight fluid seal (Figures 9A–C) and durable symptom control (in general >80% but varying rate of resolution among symptoms, studies including total of 108 patients) (78, 106, 109, 113, 124, 132, 142). Various plugging materials have been used and no material appears to demonstrate clear superiority (78, 106, 109, 113, 124, 132, 142, 143). The most commonly used materials include bone wax (78, 124, 132), bone dust (109, 113), fibrin glue, or fascia (142, 143). Most of these materials are not radiopaque and therefore a postoperative CT scan will not be useful to assess the repair. As described, assessing the fluid void (lack of fluid signal) on T2-weighted MRI scans can help determine the extent of repair (Figure 8) (102, 103). Interestingly, an experimental study in human temporal bones showed that an exceedingly small volume of bone wax (3.0–4.0 mm^2^) was needed to adequately plug a dehiscence of 1.5–3.5 mm in length via the MFC approach, and that multiple applications of bone wax resulted in extension of wax along the long axis of the superior canal into the ampulla and common crus. Extensive plugging of the defect, as shown in this model, could increase the risk of vestibular complications (141). Another study also suggested that overly-exuberant plugging may involve the common crus and lead to reduced function of both the superior and posterior canal (one reported case) (81).

**Figure 11:**
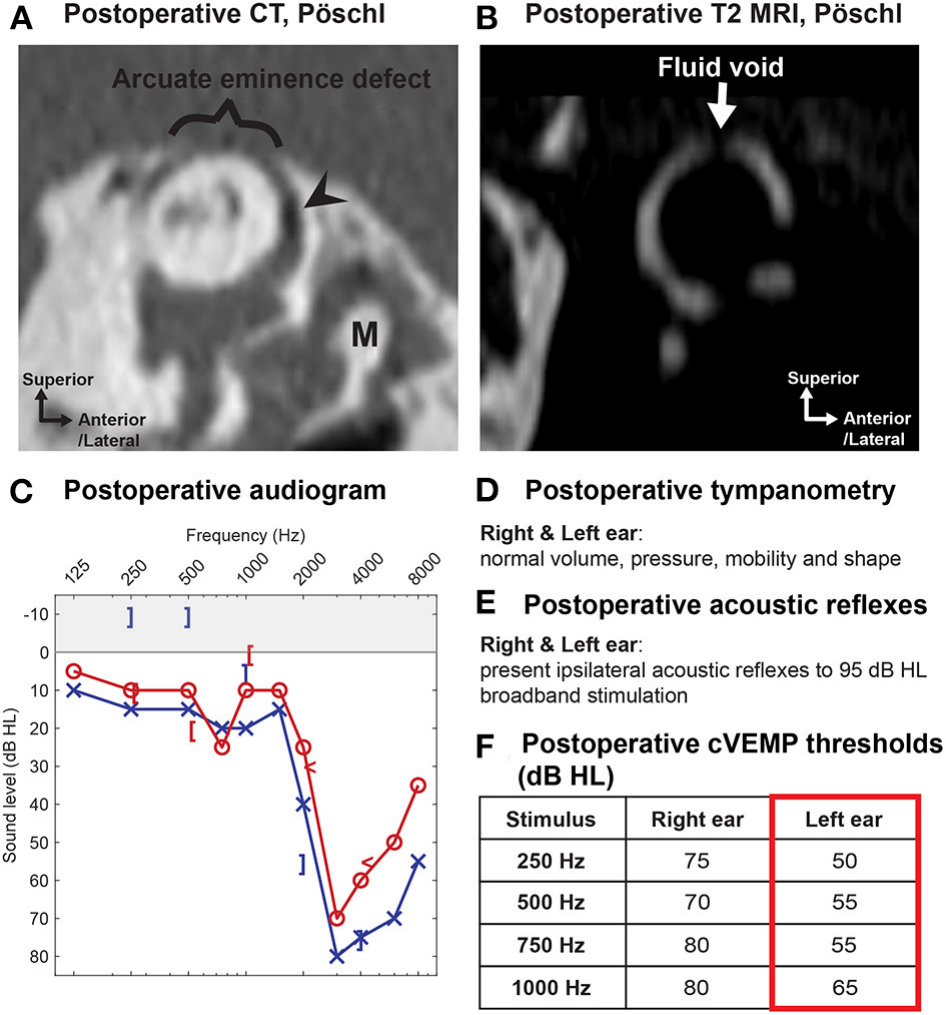
Utility of MRI in the evaluation of patients with persistent or recurrent symptoms following primary SCD repair. A 52-year old male patient underwent middle fossa craniotomy and SCD repair with resurfacing technique at the first institution. He initially experienced symptom resolution after surgery, but symptoms of left-sided aural fullness, pulsatile tinnitus, and sound-induced vertigo recurred 2 weeks later. **(A)** Postoperative (after primary SCD repair) high-resolution CT in the plane of Pöschl shows arcuate eminence defect (bracket) and focal pneumolabyrinth (arrowhead). Malleus indicated with “M.” Note that most SCD repair materials are not radio-opaque. **(B)** Postoperative T2-weighted MRI in the plane of Pöschl reveals a focal fluid void (arrow) associated with partial plugging of the superior semicircular canal that does not span the entire length of the defect. **(C–F)** Postoperative (after primary SCD repair) audiometric and vestibular testing. **(C)** Threshold audiogram reveals supranormal bone conduction thresholds (−10 dB at 250 and 500 Hz) of the left ear. **(D)** Normal tympanometry bilaterally. **(E)** Present bilateral acoustic reflexes. **(F)** cVEMP potentials demonstrate low thresholds of 50, 55, 55, and 65 dB HL in response to 250, 500, 750, and 1,000 Hz tone burst stimuli. The patient underwent revision SCD repair at the second institution with plugging of the superior canal using a transmastoid approach with stable symptom improvement 5 years after surgery.

The theory behind resurfacing techniques involves reinforcing the bone overlying the canal defect (Figure 9D). This technique has also been used with successful results (7/11 patients) (44). Resurfacing material varies and includes fascia (44), cartilage (132), bone (44), and hydroxyapatite (144). In theory, resurfacing aims to avoid occlusion of the membranous canal, thus allowing the patient to retain function of the superior canal. While some authors report maintenance of canal function following resurfacing (video head impulse testing showed normal gain in ears with SCD resurfacing repair vs. significantly reduced canal function ears with plugging, 29 ears) (132), others report decreased canal function likely associated with a partial canal plugging (1 reported case) (81), as also illustrated by the case in Figure 11. Symptom recurrence is higher with resurfacing: success rate following canal occlusion is reported >80% (studies including total of 108 patients) vs. 50–64% (42 patients) following resurfacing, perhaps due to dislocation or resorption of the graft material (44, 50, 105, 106). One study reported that symptom recurrence occurred in 4/11 patients, who underwent resurfacing, and in none of the nine patients, who underwent plugging procedure (44). Reinforcement of the resurfacing repair with hydroxyapatite, sometimes termed capping, appears to have a higher success rate than resurfacing alone, and can be performed with bone cement alone or in combination with autologous material (106, 145). A literature review comprising 13 studies and case reports found successful symptom resolution in 32/33 patients with canal plugging, 8/16 with resurfacing, and 14/15 with capping (106).

Future methods of SCD repair may include the use of customized 3D-printed prostheses and biological adhesives to preserve the superior canal lumen and canal function and to seal the defect (146). This customized, fixed-length prosthesis was designed to lock into position and occlude the bony defect (146). Refinement of the design and materials is required before translation of this concept into clinical use.

### Round Window Reinforcement

Round window reinforcement procedures have been offered by some surgeons in an effort to decrease symptoms of SCDS (147–149). The procedure has historically been used to treat symptoms associated with perilymphatic fistula (148, 149). The round window is commonly accessed by a transcanal tympanotomy approach. Stiffening of the round window is thought to dampen one of the three inner ear windows, restoring the inner ear to a non-physiologic two-window system with the oval window and the dehiscence as the remaining windows (148). In a series of 19 patients, symptom severity of autophony, sound- and pressure-induced vertigo, pulsatile tinnitus, aural fullness, and generalized disequilibrium improved following round window reinforcement with a mean improvement of two points on a seven-point scale (148). However, other reports describe a large variability in patient outcome, with some patients experiencing no resolution of symptoms or only temporary relief of symptoms (149). Furthermore, occlusion of the round window may introduce conductive hearing loss by alteration of the round window impedance (150, 151). Based on current literature, there is sparse evidence to support round window reinforcement as a viable surgery for mitigation of SCDS-related symptoms. This approach has since fallen out of favor at most centers.

## Challenges in SCDS Management

### Patients With Bilateral SCDS

Patients with symptomatic bilateral SCDS must be carefully counseled. The priorities of the clinical treatment team are to: (1) confirm that both ears with SCD are associated with localizing signs and symptoms and supporting findings on audiometric and VEMP testing (44, 78); (2) determine if there is a “worse” ear (44, 78); (3) rule out co-morbid factors such as migraines that can prolong recovery if surgery is offered, as bilateral SCD itself prolongs recovery; (4) discuss that bilateral SCDS is associated with a lower rate of complete symptom resolution (108); and (5) communicate the concerns that bilateral sequential repair could be associated with chronic balance impairment, as patients who undergo surgery bilaterally are at higher risk of vestibular hypofunction (45, 79). Another concern for some patients with bilateral radiologic SCD who undergo surgery for the only side with symptoms of SCDS is, that they may experience “unmasking” of SCD symptoms in the originally asymptomatic contralateral ear (49).

When patients have asymmetric symptoms and the more symptomatic side also demonstrates abnormal findings on audiometric and VEMP testing, selecting the surgical side is straightforward (44, 78). In a study including seven symptomatic patients with bilateral SCDS, cVEMP thresholds were lower in the more symptomatic ear, while thresholds in the contralateral ear were similar to ears without SCD (statistically significant) (125). The physical exam is also useful in these cases, as the Weber often lateralizes to the more severely affected ear in bilateral SCDS. In patients with equivocal symptoms or non-localizing signs and symptoms, the decisions for surgery and surgical side become more challenging.

Patients with bilateral SCDS report less improvement in symptoms following surgical repair compared to patients with unilateral SCDS (complete symptom resolution of primary complaint in patients with unilateral SCDS and repair: ~48%, bilateral SCDS with unilateral repair: ~12%, bilateral SCDS with sequential repair: ~20%) (108). Some studies suggest that poorer outcomes in bilateral SCDS patients may be attributable to a more generalized vestibular impairment prior to surgery and a reduced ability to compensate postoperatively, resulting in increased risk of vestibular dysfunction. One study found prolonged vestibular recovery (>4 months) in 6/11 patients with bilateral SCD and unilateral repair compared to 0/22 patients with unilateral SCD and repair) (45, 133). Also, postoperative dizziness and imbalance, and oscillopsia appear to be more prevalent in patients who undergo second-sided surgery for SCDS (~3/4 patients) (79). Preoperative vestibular testing in this cohort of patients is therefore critical prior to the first and the second surgery (if candidate for bilateral repair). This testing battery should include assessment of both the inferior and superior vestibular pathways and of semicircular canal function in all planes bilaterally, by VEMP, caloric, and VOR testing.

### Patients With Near Dehiscence Syndrome

**Figure 12:**
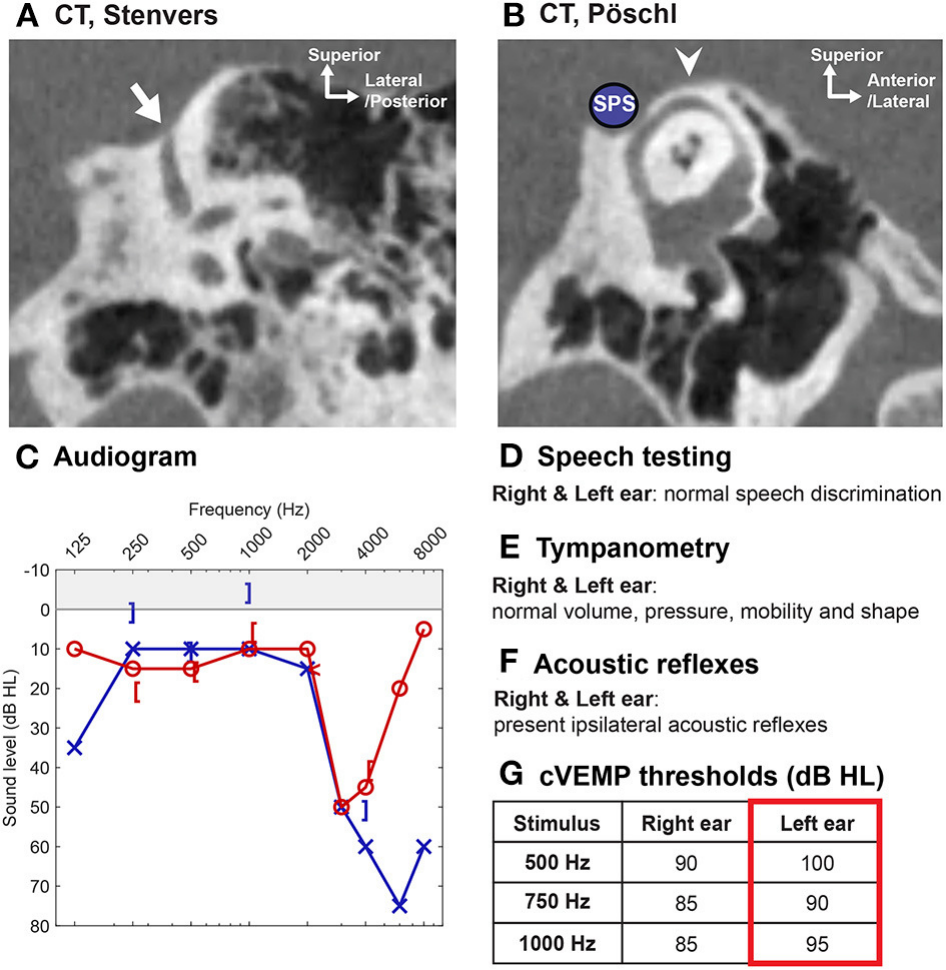
“Near dehiscence” of the superior semicircular canal. In this case, a 55-year old male presented with left-sided hearing loss, aural fullness, and vertigo. **(A)** High-resolution CT scan in the Stenvers plane with thin bone overlying the descending limb of the superior semicircular canal (arrow). **(B)** CT scan in the Pöschl view shows an intact arcuate eminence (arrowhead) and thin bone between superior semicircular canal and superior petrosal sinus (SPS, shown by blue circle). **(C–G)** Audiometric and vestibular testing confirm **(C)** a mild left-sided conductive hearing loss (high-frequency dip in thresholds not related to SCD), **(E)** normal tympanometry, **(F)** present acoustic reflex, and **(G)** normal to high cVEMP thresholds.

Patients with very thin bone (sometimes called “near dehiscence”) overlying the SSC may exhibit signs and symptoms of SCDS (Figure 12) (152). While the pathophysiology underlying this phenomenon is not entirely elucidated, several authors have argued that this variant of SCDS may reflect increased compliance of the thin bone overlying the canal or a pinpoint dehiscence (152). Indeed, pinpoint dehiscence has been found to affect inner ear acoustics in experimental cadaveric studies (12).

Diagnosing patients with near dehiscence can be challenging because the resolution of CT images does not allow one to distinguish pinpoint dehiscence vs. thin bone (as discussed previously under *Imaging—improving CT diagnosis of SCD*) (94). Several studies have shown that symptomatic patients with near dehiscence have audiometric and vestibular testing results similar to normal non-dehiscent ears and significantly different from ears with frank dehiscence, suggesting that near dehiscence does not have the same effect on inner ear biomechanics (three studies with a total of 223 SCDS ears, 90 near dehiscence syndrome ears and 83 normal ears; only one study assessed SCDS ears vs. normal ears) (65, 94, 153). By contrast, there are reports of patients with near dehiscence demonstrating supranormal bone conduction thresholds, reduced cVEMP thresholds and increased oVEMP amplitudes, suggesting that these findings may be inconsistent in this patient population (11 and 86 ears) (152, 154). ECochG has also been shown to be produce an increased SP to AP ratio among patients with near dehiscence (153). Altogether, patients with suspected near dehiscence syndrome must be carefully examined to exclude other otologic and neurotologic conditions as the condition can easily be misdiagnosed.

Repair of near dehiscence is accomplished by either reinforcing the thin bone overlying the near dehiscence or creating a small opening in the canal that may be plugged (152). In a study of 10 patients (11 ears) with near dehiscence syndrome who underwent surgical plugging and/or resurfacing, autophony improved or resolved in all cases, pulsatile tinnitus improved or resolved in 8/9 affected ears, and vertigo or disequilibrium induced by sound or pressure improved or resolved in 6/8 patients (152). Two of the 10 patients in this study suffered symptom recurrence and one patient developed unmasking of SCD symptoms in the contralateral ear (152). Of note, surgically opening the near dehiscence does not appear to worsen postoperative vestibular function (115), and complication rates have been found to be similar between surgical management of frank dehiscence and near dehiscence (insignificant difference in complication rate between 34 SCDS ears and 17 near dehiscence syndrome ears for complications including postoperative vestibular hypofunction, BPPV, posterior semicircular canal impairment and facial nerve paresis) (94).

### Management of Patients With Concurrent SCDS and Migraine

Patients with concurrent SCDS and migraine present a diagnostic and management challenge. Among patients with SCDS who undergo surgical repair, the prevalence of migraine is estimated to be 34–45% (45, 133). Several studies note that patients with concurrent SCDS and migraine appear to have prolonged recovery after surgery (45, 133). Jung et al. (133) measured the postoperative Dizziness Handicap Inventory (DHI) scores and found that more than 50% of patients with a DHI score >30 suffered from migraine (133).

It is unknown whether a pathophysiological link exists between migraine and SCD. Patients with vestibular migraine and SCDS tend to have overlapping symptoms, and thus it is hypothesized that vestibular migraine may be under-diagnosed among patients with SCDS (108). Patients with symptoms of SCDS such as generalized dizziness, imbalance, headache, and brain fog demonstrate the least degree of improvement following surgery (108). Interestingly, these patients tend to also have vestibular migraine (108).

### Management of Patients With Concurrent SCD and Otosclerosis

Patients with concurrent otosclerosis and SCD are rare, but present a diagnostic and management challenge (76, 155–161). In general, patients with concurrent otosclerosis and SCD present with conductive hearing loss, with or without SCD symptoms, absent acoustic reflexes (due to fixation of the stapes) and evidence of radiologic SCD with or without fenestral/antefenestral otosclerosis on CT (73, 74, 76, 155–158, 161). Fixation of an ossicle, stapes or malleus, reduces air-conducted sound transmission through the oval window, minimizing the occurrence of SCD symptomatology (Figure 4). Also due to decreased sound transmission, VEMP testing may have limited utility (64). While the SCD will lower VEMP thresholds, the stapes fixation will increase the thresholds, and both low, normal and high cVEMP thresholds have been observed in patients with concurrent otosclerosis and SCD (159, 161).

The largest case series of patients with concurrent otosclerosis and asymptomatic radiologic SCD described eight patients (ten ears), where seven patients (eight ears, one patient with bilateral SCD) underwent stapedotomy because SCD had not been diagnosed prior to the initial stapedotomy (76). Following stapedotomy, four patients developed unmasking of SCD symptoms. One patient did not experience unmasking of SCD symptoms, and also had near-complete closure of the ABG. Three patients (4 ears) experienced partial closure of ABG, one experienced no change and two patients had worse hearing outcome with enlarged ABG following stapedotomy (both also unmasked SCD symptoms).

For patients with concurrent SCD and otosclerosis, preoperative counseling is challenging, as undoubtedly stapedotomy carries a risk of unmasking of SCD symptoms. However, it is important to note that the true incidence of concurrent otosclerosis and radiologic SCD is unknown, as many otologists and neurotologists do not routinely obtain imaging for the work-up of conductive hearing loss and suspicion of otosclerosis (162). Current literature comprises retrospective case reports and case series, in which preoperative CT was not always obtained. In the largest case serie, 5/8 patients were diagnosed with concurrent disease because of persistent ABG after stapedotomy or unmasking of SCD symptoms, and only three were diagnosed with concurrent disease on preoperative CT. It is possible that literature is biased toward cases where ABG persisted or SCD symptoms were unmasked, which triggered CT imaging and subsequent diagnosis of concurrent otosclerosis and radiologic SCD (76, 161).

Patients who experience unmasking of SCD symptoms following stapedotomy may be candidates for surgical repair of the canal dehiscence (two cases of successful resolution of unmasked SCD symptoms have been described) (76, 156). However, multiple surgeries that involve manipulation of the inner ear could increase the risk of SNHL (127).

### Management of Children With SCDS

The occurrence of SCDS in children is rare, but there are a few reports that describe the diagnostic work-up and management of the condition in the pediatric population (163–165). The largest series included 13 children (15 ears) with radiologic SCD and symptoms of hearing loss and/or vestibular impairment (164). Ages ranged from 6 to 17 years with a mean of 11 years. Conductive or mixed hearing loss was present in seven children (nine ears). Vestibular symptoms were observed in five children and included general disequilibrium, vertigo, delayed onset of walking and other motor functions. In another series of seven children (15 ears), ranging from 5 to 11 years of age with a mean of 7 years, one child underwent surgical repair with improvement in both auditory and vestibular symptoms postoperatively (163). In a series of patients with SCDS associated the superior petrosal sinus, a 15 year old female underwent uneventful transmastoid SCD repair with durable symptom control (134). Of note, however, improvement in vestibular symptoms and stable hearing were also noted in one child at 1-year follow-up after conservative observation (163).

Behavioral observations (e.g., sudden very brief falls with immediate recovery, difficulty with or avoidance of balance-demanding activities, and delayed development of motor skills) by caregivers are important to collect, especially in younger children, when evaluating pediatric SCD, because symptom reporting is often non-specific in this patient population. Older children (typically >8 years of age) tend to report typical SCD symptoms, including autophony, amplification of bodily sounds, pulsatile tinnitus, and sound- and pressure-induced vertigo (134, 163, 164). Differences in clinical presentation of SCDS in young children and adults may warrant the development of modified diagnostic criteria for children with suspected SCDS.

Histological and radiologic studies of temporal bones have noted a higher prevalence of dehiscent and thin bone in infants and small children than in adults. One temporal bone study found that specimens from infants demonstrated uniformly thin bone over the SSC, with gradual thickening until 3 years of age (24). CT imaging studies in children (age <18 years) have demonstrated that the prevalence of radiologic near dehiscence and frank dehiscence decreases with age (25, 26). The chance of incidental SCD in young children is therefore increased compared to an adult population, and radiologic findings should be correlated with localizing signs and symptoms, audiometric testing and caregiver observations. Finally, there is currently no evidence that SCD in children is associated with other inner ear anomalies (25).

### Revision Surgery

Revision surgeries for SCDS appear to be less successful in resolving symptoms and improving quality of life compared to primary surgeries (5). In a study of 21 patients (23 ears) undergoing revision surgery for SCDS, Sharon et al. (103) found that approximately one-third of patients experienced complete symptom resolution (103). In contrast, about two-thirds of patients undergoing primary surgery for SCDS will experience complete symptom resolution (45). In both primary and revision surgeries, mechanically-explained symptoms of sound- or pressure-induced vertigo, autophony, amplification of bodily sounds, and pulsatile tinnitus are more likely to resolve than chronic disequilibrium, headaches, or fatigue (103, 108). For example, Sharon et al. found that mechanically explained symptoms resolved in 22/23 of revision cases, except for autophony which resolved in 13/17 patients, whereas a symptom like aural fullness only resolved in 7/11 patients (103).

Some case series suggest that revision surgery for SCDS carries a slightly higher risk of moderate to severe SNHL and reduced speech discrimination (two studies, 20 and 2 patients, respectively) (44, 127). Other larger studies note a similar risk of SNHL between patients undergoing primary or revision surgery (one study found no statistically significant difference in risk of profound SNHL) (103, 112). Studies may lack power to detect a difference because of small numbers. It is hypothesized that the inner ear may be sensitive to repetitive surgical trauma, and that scarring and adhesions at the surgical site may increase the trauma to the inner ear during revision surgery (103, 127). For this reason, some surgeons prefer accessing the SSC from a different approach during revision surgery (102).

Selecting appropriate candidates for revision surgery is challenging, particularly as there is some evidence of lower success rates and higher complication rates. Moreover, patients with concurrent migraine or chronic disequilibrium may present with similar symptoms. As described previously, analysis of the fluid void of the SSC using T2-weighted MRI imaging can be used to evaluate for residual canal dehiscence following primary surgical repair (Figures 8, 11) (102).

The utility of oVEMP and cVEMP testing in assessing candidacy for revision surgery appear to be limited because SCD effects on VEMP can be obscured by peripheral vestibular deficits following the primary SCD repair (102, 103). One study found that only 4/17 patients with unresolved/recurrent symptoms had elevated oVEMP amplitudes after primary repair/before revision surgery (103) and another study demonstrated low cVEMP thresholds in 4/9 revision surgery candidates (102). However, normalization of VEMP thresholds after successful primary repair (and revision repair) has been reported, which suggests that continued low threshold (high amplitude) VEMP indicates unsuccessful repair (two studies, total of nine patients all with normalization postoperatively) (118, 166). It is possible that VEMP thresholds are less sensitive following initial surgical manipulation and may not change following revision surgery (103).

## Future Considerations

While tremendous progress has been made over the past two decades in the diagnosis and management of SCDS, there are a number of important research questions that are still unanswered.

First, the etiology and pathophysiology of SCDS are incompletely understood. There are a wide range of vestibular and auditory symptoms, as well as symptom severity, among SCDS patients that does not always correlate with size and location of the defect. Additionally, some patients may have developed maladaptive behaviors and cognition in response to ongoing symptoms, which complicates symptom presentation.

Second, contemporary diagnostic measures such as audiometric and VEMP testing do not fully capture changes in inner ear biomechanics among patients with SCD, and atypical signs and symptoms, near dehiscence, bilateral dehiscence, and determining candidates for revision repair pose diagnostic challenges. Studies investigating novel diagnostic methods independent of innate vestibular or auditory function are important in solving these challenges.

Third, surgical needs include the ability to create a durable tight fluid seal like SCD plugging but without affecting fluid motion of the SSC, and to reduce the associated complications including dizziness and hearing loss. Customized 3D-prostheses may represent a future approach (146).

**Figure 13:**
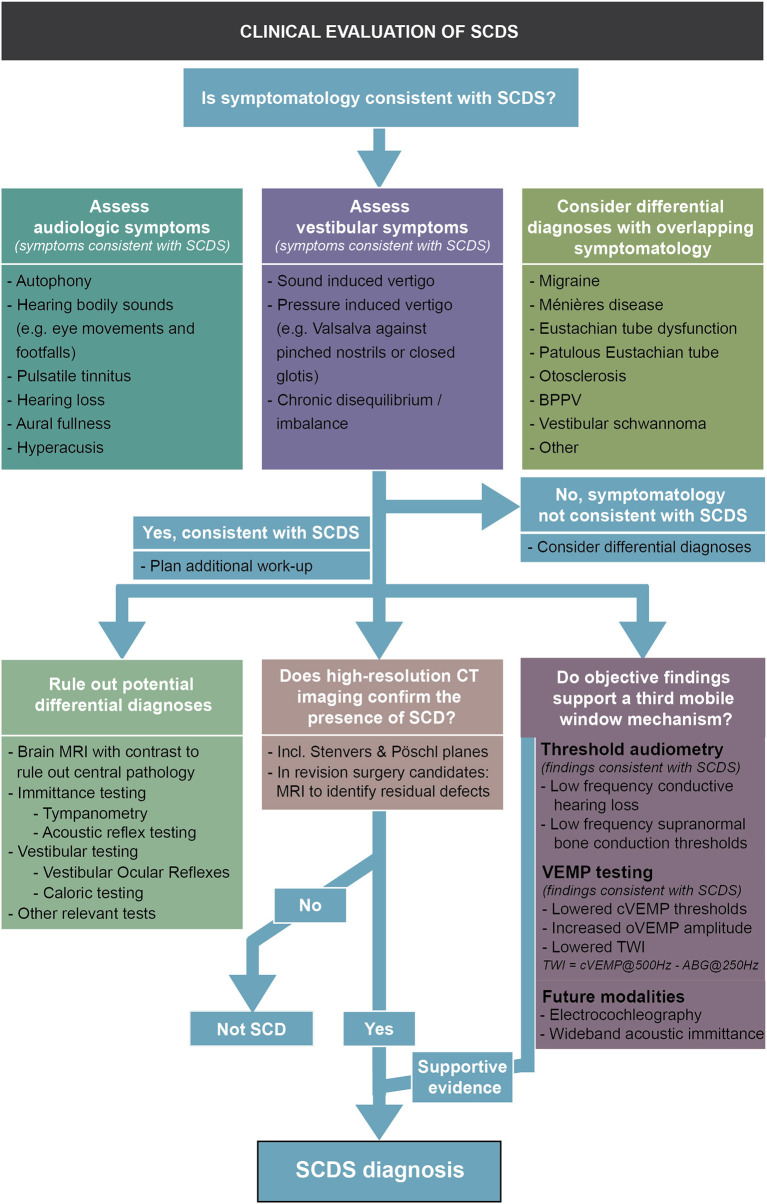
SCD diagnostic algorithm. Evaluation scheme to guide the clinician through a thorough and complete clinical evaluation of a potential SCDS patient.

Large cohort studies comparing surgical approaches are lacking, in part due to the rarity of the disease, but also due to high variability in technique among surgeons. Additionally, a disease-specific outcome measure in SCDS has not been identified. As current studies rely on a variety of outcome measures, comparing results among studies is challenging. Developing a consensus on the diagnostic criteria and outcome measures is critical to allow clinical outcomes research of SCDS to progress forward. In Figure 13, we proposed an evaluation scheme to guide the clinician through a thorough and complete clinical evaluation of a potential SCDS patient.

## Conclusions

SCD has been increasingly recognized as a treatable cause of vestibular and auditory dysfunction. Remarkable strides have been made in understanding the pathophysiology of this unusual third window condition. Improvements in CT resolution as well as more widespread supranormal bone conduction threshold testing, coupled with refinements in cervical and ocular VEMPs have improved the diagnostic yield in the evaluation of patients with a suspected third window. Temporal bone MRI is a valuable imaging modality in the assessment of the patient with a new SCDS diagnosis or in the evaluation of a patient who may be a candidate for revision surgery. WAI and ECochG have been investigated as novel measures to assess SCD biomechanics. Operative management of SCDS has seen advances in the use of minimally invasive surgical corridors, skull base endoscopy, and a variety of repair materials, although debates persist about the optimal surgical approach, technique, and material. Plugging of the defect, rather than resurfacing, is associated with longterm symptom control in most cases. Finally, comparative outcome studies are needed to assess challenging cases, such as patients with bilateral dehiscence, near dehiscence, revision cases, and concurrent SCDS and migraine disorder.

## Author Contributions

KE and DL concepted the review. KE drafted the manuscript. KE, DC, HN, MK, PC-T, and DL critically revised the manuscript. All authors gave final approval.

## Conflict of Interest

The authors declare that the research was conducted in the absence of any commercial or financial relationships that could be construed as a potential conflict of interest.
